# The impact of introducing ambulance and delivery fees in a rural hospital in Tanzania

**DOI:** 10.1186/s12913-021-06107-x

**Published:** 2021-01-28

**Authors:** Corinna Vossius, Estomih Mduma, Robert Moshiro, Paschal Mdoe, Jan Terje Kvaløy, Hussein Kidanto, Sara Lyanga, Hege Ersdal

**Affiliations:** 1 Critical Care and Anaesthesiology Research Group, Stavanger University Hospital, PB 8100, 4068 Stavanger, Norway; 2 Haydom Global Health Research Center, Haydom Lutheran Hospital, Haydom, Tanzania; 3 Department of Research, Stavanger University Hospital, Stavanger, Norway; 4 Faculty of Health Sciences, University of Stavanger, Stavanger, Norway; 5 Muhimbili National Hospital, Dar es Salaam, Tanzania; 6 Department of Mathematics and Physics, University of Stavanger, Stavanger, Norway; 7 Medical College, Aga Khan University, Dar es Salaam, Tanzania

**Keywords:** Birth attendance, Low resource setting, Maternal health, Newborn health, Delivery fees, Labour complications, Pregnancy complications, Perinatal survival

## Abstract

**Background:**

Access to health care facilities is a key requirement to enhance safety for mothers and newborns during labour and delivery. Haydom Lutheran Hospital (HLH) is a regional hospital in rural Tanzania with a catchment area of about two million inhabitants. Up to June 2013 ambulance transport and delivery at HLH were free of charge, while a user fee for both services was introduced from January 2014. We aimed to explore the impact of introducing user fees on the population of women giving birth at HLH in order to document potentially unwanted consequences in the period after introduction of fees.

**Methods:**

Retrospective analysis of data from a prospective observational study. Data was compared between the period before introduction of fees from February 2010 through June 2013 and the period after from January 2014 through January 2017. Logistic regression modelling was used to construct risk-adjusted variable-life adjusted display (VLAD) and cumulative sum (CUSUM) plots to monitor changes.

**Results:**

A total of 28,601 births were observed. The monthly number of births was reduced by 17.3% during the post-introduction period. Spontaneous vaginal deliveries were registered less frequently with a decrease of about 17/1000 births in non-cephalic presentations. Labour complications and caesarean sections increased with about 80/1000 births. There was a reduction in newborns with birth weight less than 2500 g. The observed changes were stable over time. For most variables, a significant change could be detected after a few weeks.

**Conclusion:**

After the introduction of ambulance and delivery fees, an increase in labour complications and caesarean sections and a decrease in newborns with low birthweight were observed. This might indicate that women delay the decision to seek skilled birth attendance or do not seek help at all, possibly due to financial reasons. Lower rates of births in a safe health care facility like HLH is of great concern, as access to skilled birth attendance is a key requirement in order to further reduce perinatal mortality. Therefore, free delivery care should be a high priority.

## Background

The United Nations’ 2030 Agenda for Sustainable Development third goal aims to “Ensure healthy lives and promote well-being for all at all ages”, and the sub-goals 3.1, 3.2 and 3.4 ask specifically to reduce mother, newborn and premature mortality, respectively [1]. Skilled birth attendance is one of the high impact interventions to improve maternal and newborn health [2]. Thus, access to health care facilities with trained personnel is a key requirement to enhance safety for mothers and newborns during labour and delivery.

Haydom Lutheran Hospital (HLH) is a regional hospital in rural Northern-central Tanzania with a catchment area of about two million inhabitants. It serves as a referral hospital, receiving delivering women from three district hospitals, two health centers, several dispensaries, and from home [3]. On average, women in this region give birth to five children, the first pregnancy being at a median age of about 20 years. Overall, in Tanzania, 63% of births occur in a health facility, primarily in public sector facilities, and 36% of births occur at home. Women with no education, those living in rural areas, and those in the poorest households are the most likely to deliver at home, as indicated for the catchment area of HLH with a proportion of about 50% home deliveries [3, 4].

As HLH is located in a rural area with a population of low social-economic status and limited public transport, a hospital driven ambulance service was introduced in 2008, offering free transport for patients including parturient women. All hospital services connected to child delivery were also free of charge. However, due to a worse financial situation at HLH several user fees were introduced in 2013/2014, and since July 2013 a fee of about 1 USD (2000 TZs) per each kilometer distance has been charged for ambulance services, and since January 2014 women have been charged about 12 USD (25,000 TZs) for a vaginal delivery and about 30 USD (60,000 TZs) for a caesarean section (CS) unless it was proved that the family was not able to afford the cost.

In 2009, HLH was chosen as one of eight study sites to implement the Helping Babies Breathe (HBB) program, designed to improve birth attendants’ skills through simulation training [5]. HBB training was introduced at the Maternity Unit, HLH, in February 2010, and later a continuous quality improvement program was deployed. Simultaneously, a unique research infrastructure was established, collecting data from every birth [6, 7].

Recently, we reported outcomes of this HBB quality improvement program, from February 2010 through January 2017. Besides an increased perinatal survival, we observed an increase in pregnancy and labour complications as well as CSs in the period after the introduction of ambulance and delivery fees, while the total number of deliveries at HLH decreased [8]. We suspect that the introduction of fees might impact the women’s and/or respective families’ decision to seek birth attendance at HLH, resulting in a lower number of births and a higher proportion of women with pregnancy and labour complications. Furthermore, we suspect that the decision to seek help might be delayed due to the financial burden, resulting in a higher number of labour complications and hence CSs.

The aim of this study was to explore the impact of introducing user fees on the population of women giving birth at HLH, regarding the total number of births and factors indicating potential high-risk deliveries, in order to document potentially unwanted consequences in the period after introduction of fees.

## Methods

### Setting

This study represents a retrospective analysis of data collected in a prospective observational study conducted at HLH from February 2010 and still ongoing. HLH provides comprehensive emergency obstetric and basic newborn care on a 24/7 basis, including six delivery rooms with one delivery bed each, and one operating theatre where CSs take place. Data were collected from all delivery rooms and the operating theatre [6, 7].

### Data collection and variables analysed

Data collection was performed by trained research assistants who observed every delivery in the labour ward and has previously been described in detail [6, 7]. Data collection for this study took place from February 2010 through January 2017, but data collected between 1.7.2013 (introduction of ambulance fee) and 01.01.2014 (additional introduction of delivery fees) were not included into the analysis. Data were collected prospectively during this study period, and there was a data quality control system to ensure the validity. Information collected included pregnancy complications, labour process and outcome, newborn information, and birth attendant information. For this study we only looked at factors describing perinatal characteristics and risk-factors not related to clinical management. In detail, the following variables were analysed: number of births per month, multiple births, antenatal care, gestational age, birth weight, fetal presentation (cephalic or non-cephalic), and macerated stillbirths. In addition, we included the following variables which are partly related to clinical management: spontaneous vaginal deliveries (SVD), fetal heart rate status on admission, and labour complication comprising obstructed labour, vacuum extraction, CS, pre-eclampsia/eclampsia, bleeding before birth, uterine rupture, and cord prolapse.

Analysis of gestational age revealed a large inter-rater variability before and after the introduction of delivery fees due to training of the healthcare workers in estimating gestational age in 2013, and the variable was thus discarded from the analyses.

### Statistical analysis

Data was compared between the period before introduction of fees from February 2010 through June 2013 (41 months) and the period after introduction of fees from January 2014 through January 2017 (37 months).

Count data are presented as numbers and percentages and continuous data as means and standard deviations. Potential indicators for high-risk deliveries in the period before respectively after the introduction of fees were compared by either Chi-square test or Student’s t-test, as appropriate.

To detect and quantify potential changes in proportions of high-risk deliveries at HLH in the period after the introduction of fees, we constructed a Variable Life Adjusted Display (VLAD) plot [9], presenting the cumulative sum of observed numbers for each variable in the post-introduction period, minus the expected numbers if the situation without introduction of fees had persisted. The VLAD plot can then be interpreted as the cumulative excess or deficiency of risk factors over time, compared to the pre-introduction period. Variables included into the VLAD-plot were selected due to statistically significant differences in the period before versus after introduction of fees and to clinical relevance. The VLAD-plot comprised the following variables: total number of births, abnormal and not measured fetal heart rate status on admission, fetal presentation other than cephalic, SVD, labour complications, CSs, birth weight below 2500 g, and birth weight above 4000 g.

To verify statistical significance of the findings of the VLAD-plots and quantifying when enough information to claim a significant change had been accumulated, corresponding cumulative sum (CUSUM) plots were also constructed [10, 11]. These plots have a formal signal limit where the process is deemed to have demonstrated a persistent change when the signal limit is crossed. These CUSUM plots were constructed such that they were able to detect an increase in the proportion for those variables showing an increasing tendency and a decrease for those variables showing a reduction. The signal limits where chosen such that the processes would give a false alarm (type I error) at most once per 10 years on average.

### Ethical considerations

This study was approved by the National Institute for Medical Research (NIMR), Ministry of Health in Tanzania (the HBB CQI program Ref. NIMR/HQ/R.8a/Vol.IX/1247 and the Safer Births project Ref. NIMR/HQ/R8a/Vol. IX/1434), and by the Regional Committee for Medical and Health Research Ethics, Western Norway (Ref. 2009/302 and Ref number 2013/110/REK). Data is not openly available, and we received permission by NIMR to access the raw data for this study and to publish the findings. All relevant health care providers were informed about the different HBB CQI and Safer Births quality assessment studies and gave oral consent. Patients were also informed about ongoing studies, but consent was not required for this descriptive study.

## Results

A total of 28,601 births were included; 16,375 births before the introduction of fees and 12,226 after.

Table 1 compares indicators for high-risk deliveries before and after the introduction of fees. The monthly number of births was reduced by 17.3% during the post-introduction period, and there was a lower share of maternal infections, non-cephalic presentations and SVD. Normal fetal heart rates were registered less frequently, while labour complication and CSs were more frequent, as well as not-measured fetal heart rates. There was a general increase in mean birth weight, and in more detail, a reduction of newborns with birth weight less than 2500 g and an increase in newborns weighing more than 4000 g. In newborns weighing more than 4000 g a CS was performed in 28.4% in the period before the introduction of fees and in 71.6% in the period after.

**Table 1:** Comparison of indicators for high-risk deliveries before and after the introduction of delivery fees, all variables included

Variable^a^	Before 1.2.10–30.6.13	After 1.1.14–31.1.17	P	Missing data^b^
Number of births, total	16,375	12,226		
Number of births per month, mean (CI)	399 (390–409)	330 (317–344)	< 0.001	
No antenatal care visit	122 (0.7 (0.6–0.9))	133 (1.1 (0.9–1.3))	0.002	2 (0)
Multi pregnancy	553 (3.6 (3.3–3.9))	483 (4.0 (3.6–4.3))	0. 108	933 (3.3)
Presentation other than cephalic^c^	1077 (6.6 (6.2–7.0))	584 (4.8 (4.4–5.2))	< 0.001	8 (0)
Birth weight in gr, mean (CI)	3099 (3092–3107)	3277 (3268–3287)	< 0.001	374 (1.3)
Birth weight < 2500 g	1301 (8.0 (7.5–8.4))	769 (6.3 (5.9–6.7))	< 0.001	0
Birth weight > 4000 g	385 (2.4 (2.1–2.6))	718 (5.9 (5.5–6.3))	< 0.001	0
Gestation week, mean (CI)	36.4 (36.4–36.5)	38.4 (38.4–38.5)	Discarded from analysis	1052 (3.7)
Macerated stillbirth	185 (1.1 (1.0–1.3))	157 (1.3 (1.1–1-5))	0.157	0
Spontaneous vaginal delivery	12,732 (82.2 (81.7–82.9))	9159 (74.9 (74.1–75.7))	< 0.001	891 (3.1)
Labour complications	2525 (15.4 (14.9–16.0))	2895 (23.7 (22.9–24.4))	< 0.001	0
- Bleeding before labour	86 (0.5 (0.4–0.6))	88 (0.7 (0.6–0.9))	0.036	
- Pre-Eclampsia/Eclampsia	58 (0.4 (0.3–0.4))	66 (0.5 (0.4–0.7))	0.018	
- Obstructed labour	868 (5.3 (5.0–5.6))	548 (4.5 (4.1–4.8))	0.002	
- Vacuum extraction	130 (0.8 (07.-0.9))	25 (0.2 (0.1–0.3))	< 0.001	
- Uterine rupture	17 (0.1 (0.1–0.2))	24 (0.2 (0.1–0.3))	0.056	
- Cord prolaps	93 (0.6 (0.5–0.7))	94 (0.8 (0.6–0.9))	0.037	
- Caesarean section	2329 (14.2 (13.7–14.8))	2808 (23.0 (22.2–23.7))	< 0.001	
Fetal heart rate status on admission abnormal or not measured	1085 (6.6 (6.2–7.0))	2308 (18.9 (18.2–19.6))	< 0.001	2 (0)
- Abnormal	461 (2.8 (2.6–3.0))	569 (4.7 (4.3–5.0))	< 0.001	
- Non-detectable	310 (1.9 (1.7–2.1))	231 (1.9 (1.6–2.1))	0.920	
- Not measured	314 (1.9 (1.7–2.1))	1508 (12.3 (11.8–12.9))	< 0.001	

^a^If not otherwise stated, data is presented as n (% with CI)

^b^Some events were only registered when occurring and hence have low missing data

^c^Breech, shoulder dystocia, transverse, other

**Fig. 1:**
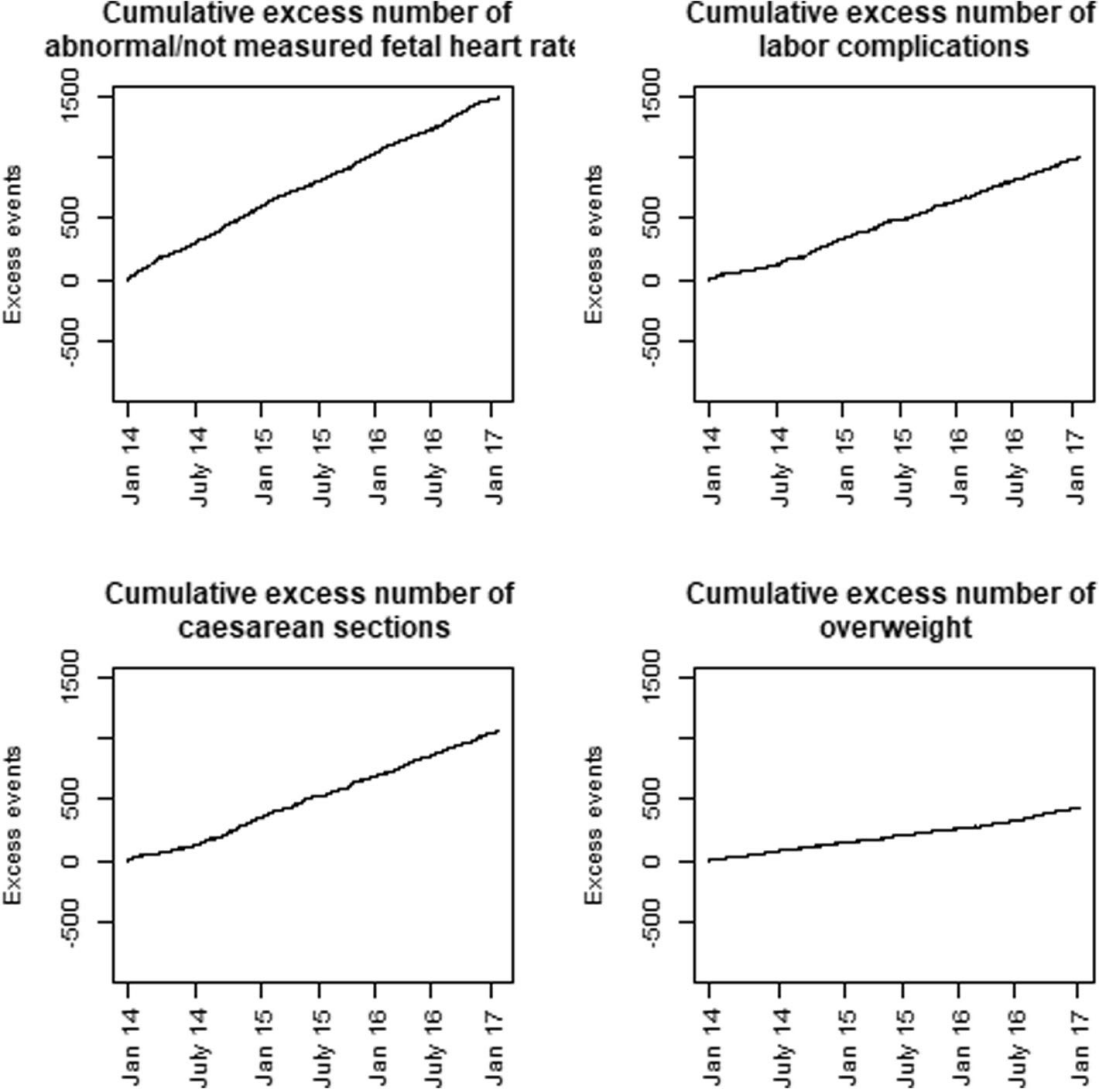
Variable Life Adjusted Display (VLAD) plots presenting cumulative excess of indicators with increasing numbers in the post-introduction period

**Fig. 2:**
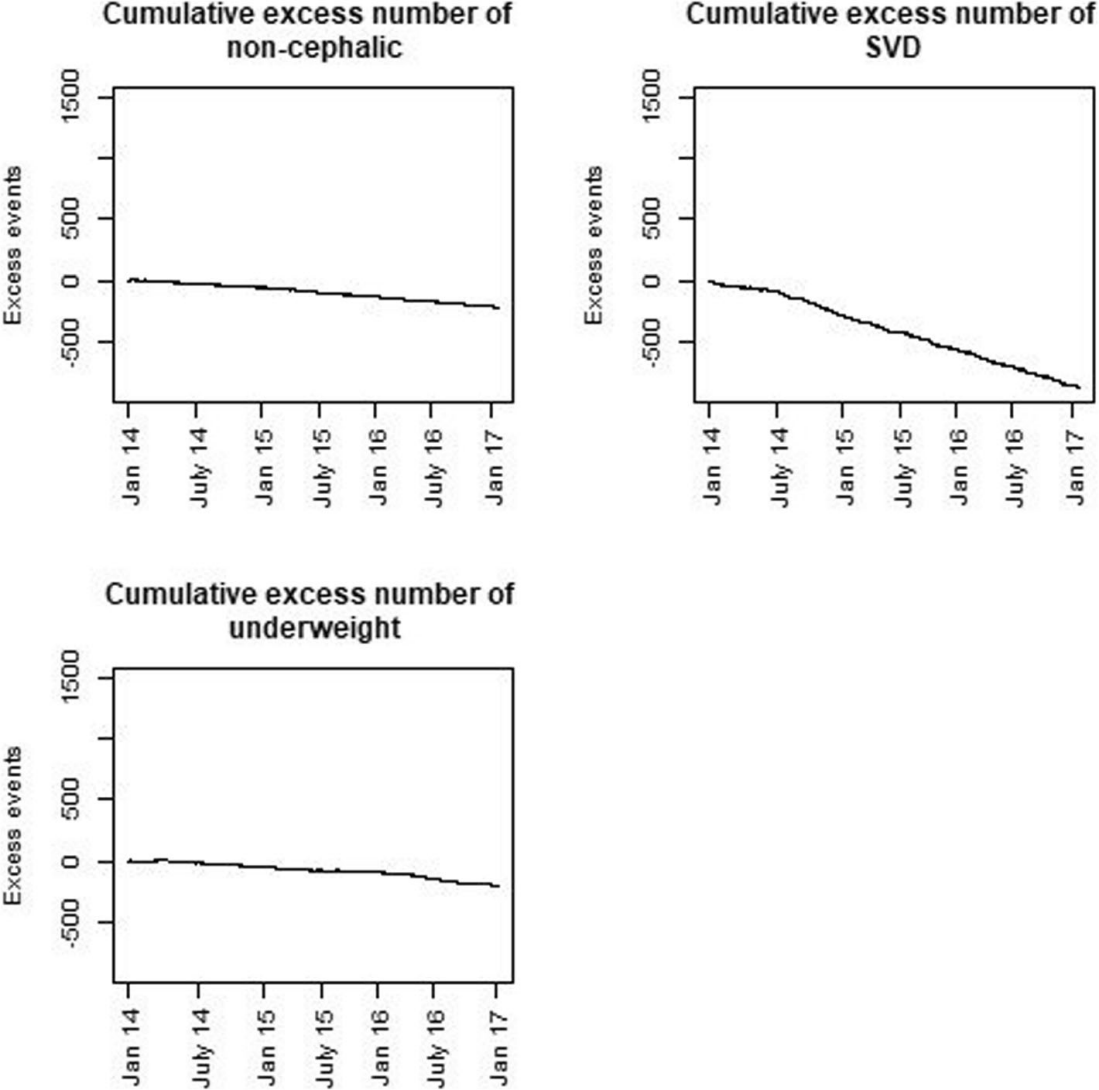
Variable Life Adjusted Display (VLAD) plots presenting the cumulative deficiency of indicators with decreasing numbers in the post-introduction period

Results of the VLAD plots are presented in Figs. 1 and 2. In the 37 months period after the introduction of fees there was an excess of births with abnormal or not-measured fetal heart rates of about 1500, and approximately 1000 excess complicated labours including CSs. Simultaneously, the number of SVDs was reduced by about 800, and a slight decrease (approximately 200) in births with non-cephalic presentation was noted. Furthermore, there was approximately 450 more newborns with birthweight above 4000 g (Fig. 1), and about 200 less newborns with birthweight below 2500 g (Fig. 2). The observed increases and decreases were stable over time.

**Fig. 3:**
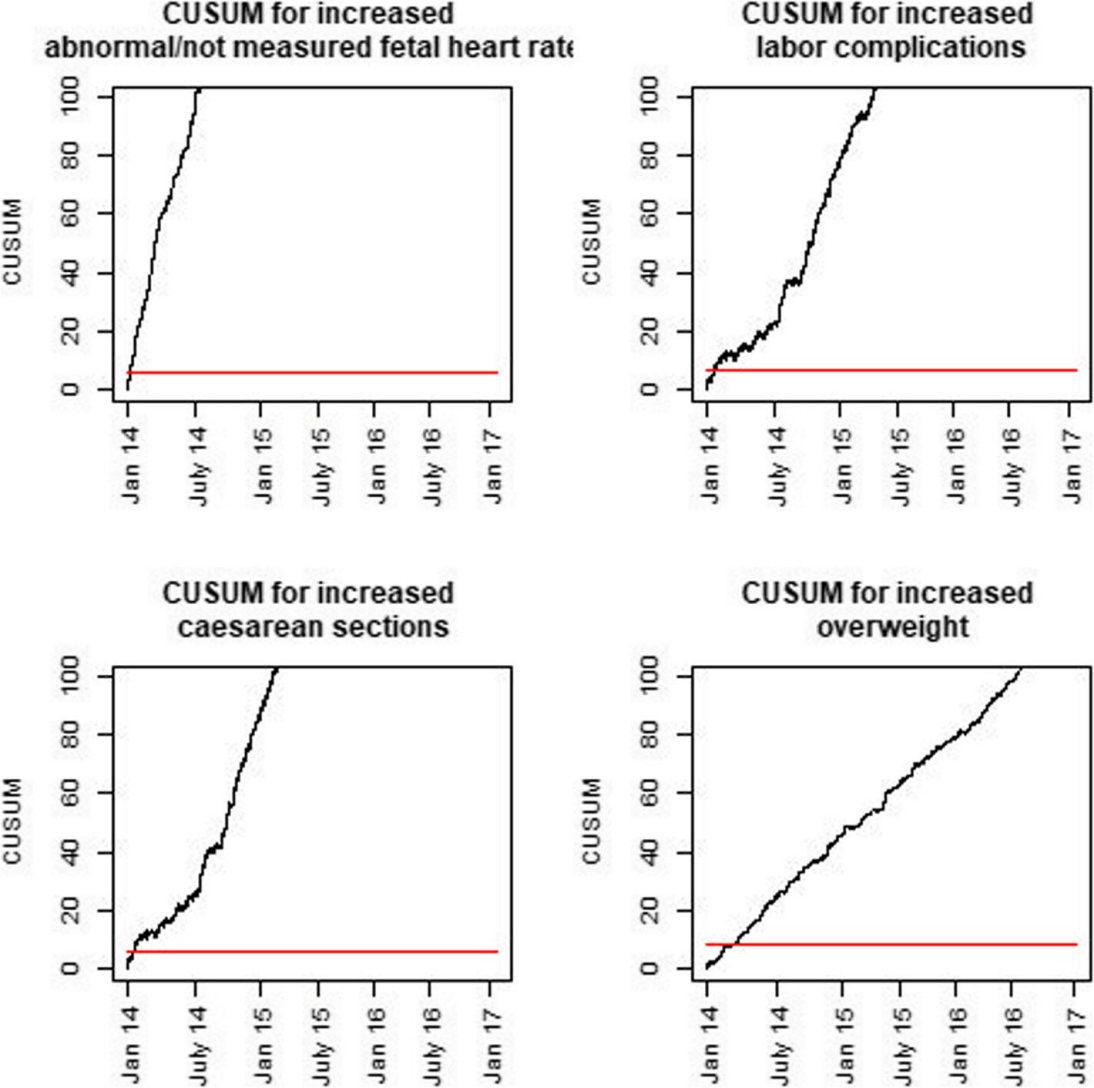
Cumulative sums (CUSUM) of indicators with increasing numbers in the post-introduction period

**Fig. 4:**
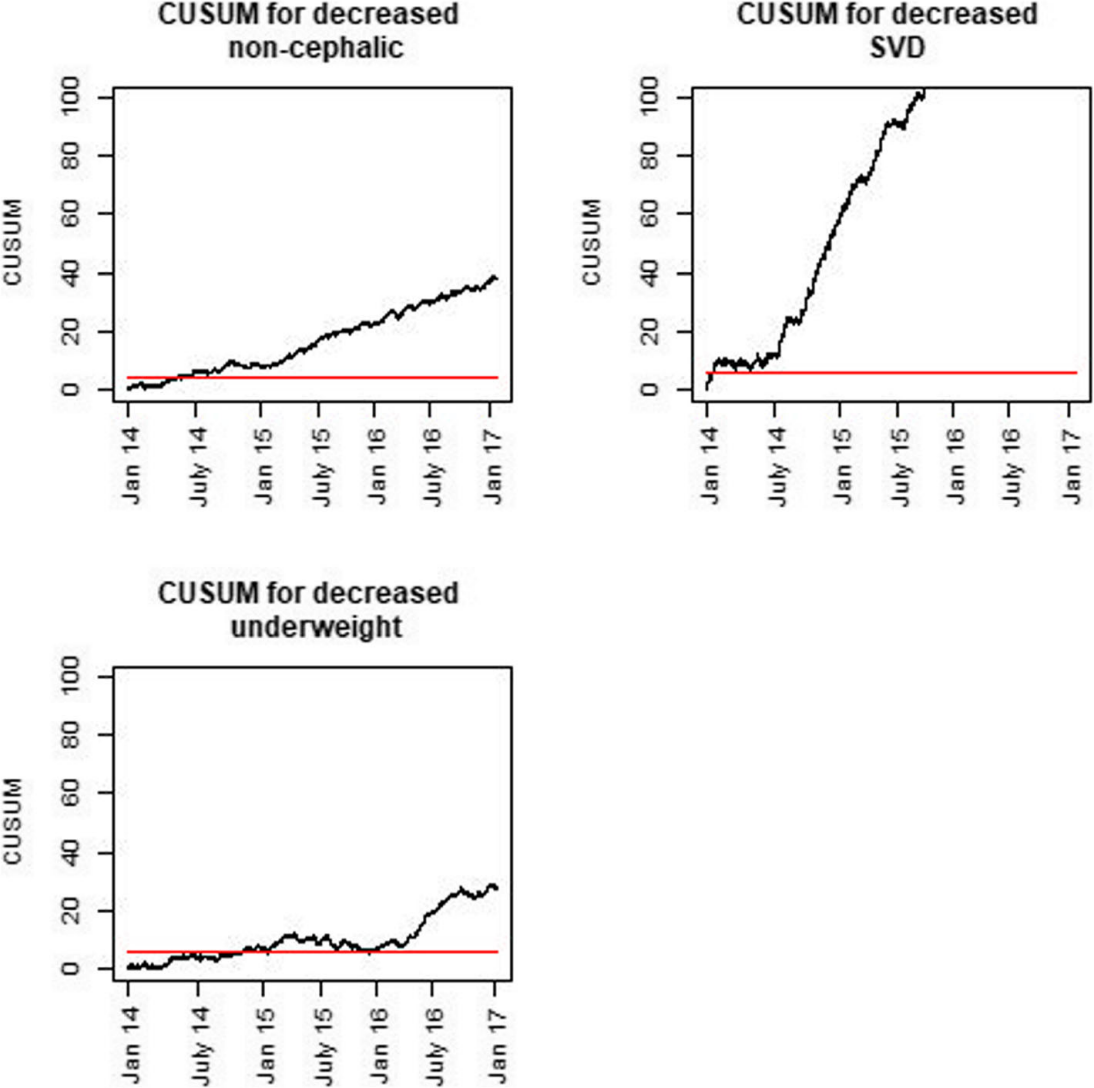
Cumulative sums (CUSUM) of indicators with decreasing numbers in the post-introduction period

The CUSUM plots indicate that the observed changes became statistically significant during the first year after the introduction of fees (Figs. 3 and 4). For most variables, a significant change could be detected after a few weeks.

## Discussion

This study explores the impact of introducing user fees on the population of women giving birth at a rural referral hospital in Tanzania. We report an overall decline in births of 17.3% during the period after introduction of the fees, while the overall birth rate in Tanzania decreased by 3.7% between 2010 and 2016 [4]. Concurrently, an increase in labour complication, CSs and newborns with birthweight above 4000 g were noted, and a decrease in SVDs, births with non-cephalic presentation and newborns with birthweight below 2500 g. These changes reached a statistically significant threshold already a few weeks after the introduction of fees.

Our findings indicate that less women chose to deliver in this hospital, possibly as a consequence of increased out-of-pocket contributions. Unfortunately, we do not have information whether delivery alternatively took place at one of the other health facilities in the region or at home. However, in parallel with the overall decline in number of births at HLH, we observed a change in the spectre of births with an increase in factors indicating more high-risk deliveries. The significant increase in abnormal fetal heart rate assessments on admission indicates that women arrived later in labour. A concurrent raise in recorded labour complication, cephalic presentation and newborns with a higher birthweight resulting in more CSs suggest a larger share of asphyxia-related complications, potentially related to obstructed or prolonged labour, in the period after introduction of the fees. Unfortunately, as shown in Table 1, data collection failed to register the cause for performing a CS in more than 60% of all CSs. Still, this probably indicates that women delayed seeking help at HLH in order to avoid paying fees but eventually came when serious labour complications had developed and were obvious. This hypothesis is also supported by the increased share of births where fetal heart rate was not measured on admission, likely due to the need for immediate actions. Moreover, less low birthweight newborns were born in the hospital after introduction of the fees, indicating that the population did not seek referral hospital delivery for these early births.

We know that low education, living in rural areas, and poverty indicate an increased risk for home delivery [4]. Further, unforeseen out-of-pocket payments might have devastating consequences for the families’ finances, and transportation fees represent an important factor affecting the decision to seek professional medical help [12–14]. In Tanzania in 2011/12, 44% of the population had less than 1.25 USD per day, with even more prevalent poverty in rural districts [15]. In this perspective, fees of 12–30 USD are considerable and a great burden to the family economy. Previous studies from various sub-Saharan countries could show that the removal of delivery fees resulted in an increase of facility-based deliveries, and a study from Lesotho showed that the removal of ambulance and delivery fees resulted as well in a decrease in neonatal and maternal mortality rates [16–20]. The findings of this study implicate that an excess of about 80 CSs per 1000 births as a consequence of user fees might have been avoided by more timely birth attendance. At the same time the number of high-risk deliveries like non-cephalic presentations and premature births decreased by about 17 per 1000 births each, indicating that these mothers with newborns in great need for skilled birth attendance either had no access to, did not understand the risk-situation, or did not want to seek medical help. The only risk-group choosing more frequently to deliver at HLH were mothers of newborns with a high birthweight, but a 2.5-fold increase in CSs for this group indicates a tendency to delay seeking help as well in these cases.

### Strengths and limitations

The major strengths of this study are the prospective and detailed collection of data over a study period of seven years, and the high number of observed cases, performed in a rural setting of a low-income country with a high burden of disease.

As a main weakness there were several research and administrative exposures during the study period that might act as confounders to our findings, i.e. implementation of the HBB program, several randomized controlled studies, and a high turn-over of midwives [8]. We therefore aimed at only including variables into our analyses that would be independent from in-hospital care. Variables like the number of CSs and assessment of fetal heart rate on admission might be partly impacted by the quality of care, and thus not only reflecting family decisions to seek medical help at HLH. However, the high number of cases and the stability of the changes over time indicate that our findings are robust and can be attributed to the introduction of fees. Nevertheless, being an observational study, residual confounding is likely. A population-based birth registry including all deliveries in the catchment area, and providing as well information about birth attendance in home deliveries, would have given a complete picture, but this is not available.

Since the study was done in a single centre, our findings cannot be generalized without precaution.

## Conclusion

After the introduction of ambulance and delivery fees at HLH we observed an increase in labour complications and CSs of about 80 CSs per 1000 births. This might indicate that women delay the decision to seek skilled birth attendance until labour complications have occurred. In addition, we observed a decrease in non-cephalic presentations and newborns with low birthweight of about 17 per 1000 births each, indicating that some mothers in great need for skilled birth attendance either had no access to or did not want to seek medical help, possibly due to financial reasons.

Lower rates of births in a safe health care facility like HLH is of great concern, as access to skilled birth attendance is a key requirement in order to further reduce perinatal mortality. Therefore, free delivery care should be a high priority.

## Data Availability

The datasets used and analysed during the current study are available from the corresponding author on reasonable request.

## Abbreviations

CS: Caesarean section
CUSUM: Corresponding cumulative sum
HBB: Helping Babies Breathe
HLH: Haydom Lutheran Hospital
SVD: Spontaneous vaginal deliveries
VLAD: Variable Life Adjusted Display
